# A237 VIRULENCE POTENTIAL OF BACTERIA ISOLATED FROM NON-INFLAMED COLON IN PATIENTS WITH INFLAMMATORY BOWEL DISEASES

**DOI:** 10.1093/jcag/gwab049.236

**Published:** 2022-02-21

**Authors:** N Arjomand Fard, M Bording-Jorgensen, J Webb, S Veniamin, T Perry, E Wine

**Affiliations:** 1 University of Alberta, Edmonton, AB, Canada; 2 Pediatrics, University of Alberta, Edmonton, AB, Canada

## Abstract

**Background:**

As inflammation can impact microbes, our lab has focused on non-inflamed bowel sections of patients with inflammatory bowel diseases (IBD) and found bacterial alterations in the terminal ileum. We are also interested in the appendix given the involvement of the peri-appendicular region and effects of appendectomy in ulcerative colitis.

**Aims:**

In this study, we aimed to identify and evaluate microbes from the appendix and non-inflamed regions of the colon in IBD patients. We hypothesized that microbes originating from the non-inflamed sections of the colon or appendix are more invasive and could trigger inflammation in IBD patients.

**Methods:**

16S DNA Sanger sequencing was performed on bacteria, aerobically and anaerobically cultured from tissues of the appendix, peri-appendicular region, cecum, and ascending colon, collected from the resected colons of 5 pediatric IBD patients. The invasive capacity of the identified bacteria was evaluated by gentamicin protection assays on Caco-2 intestinal epithelial cells *in vitro*. Presence of select invasive or adhesive genes in the bacteria was assessed by PCR.

**Results:**

*Escherichia coli* was the most frequently cultured species in the aerobic cultures; additional aerobic species included *Enterococcus* and *Klebsiella*. *Bifidobacterium* and *Erysipelatoclostridium* were identified among the anaerobic cultures. Gentamicin protection assays indicated that *Klebsiella* isolated from the peri-appendicular region was significantly more invasive (mean ~3000 CFU/ml) than HB101 (~1000 CFU/ml; non-invasive control; P<0.05). *Enterococcus avium* and *Enterococcus faecalis* were also more invasive than HB101 in the peri-appendicular region (~10000 CFU/ml) and cecum (~4000 CFU/ml; P<0.05), respectively. *E. coli* isolated from the appendix showed a higher invasive potential (~3000 CFU/ml) than *E. coli* isolated from other sections. Additionally, PCR showed that *E. coli* obtained from different sections, except the ascending colon of one the patients, had the *fimH* gene while the other types of bacteria did not. None of the isolated bacteria had Hemolysin ( *hlyA*) or attaching and effacing ( *eaeA*) genes.

**Conclusions:**

Bacteria, especially *E. coli*, from non-inflamed bowel in IBD (including the appendix) appear to have increased invasive potential. This suggests that the microenvironment in these regions could be altered, resulting in increased invasion of bacteria or the gut harboring more invasive microbial populations in IBD patients. As a result, inflammation could be triggered or exacerbated through this reservoir of pathobionts.

**Funding Agencies:**

CIHR

